# Keeping Signals Straight: How Cells Process Information and Make Decisions

**DOI:** 10.1371/journal.pbio.1002519

**Published:** 2016-07-18

**Authors:** Michael T. Laub

**Affiliations:** Howard Hughes Medical Institute, Department of Biology, MIT, Cambridge, Massachusetts, United States of America

## Abstract

As we become increasingly dependent on electronic information-processing systems at home and work, it’s easy to lose sight of the fact that our very survival depends on highly complex biological information-processing systems. Each of the trillions of cells that form the human body has the ability to detect and respond to a wide range of stimuli and inputs, using an extraordinary set of signaling proteins to process this information and make decisions accordingly. Indeed, cells in all organisms rely on these signaling proteins to survive and proliferate in unpredictable and sometimes rapidly changing environments. But how exactly do these proteins relay information within cells, and how do they keep a multitude of incoming signals straight? Here, I describe recent efforts to understand the fidelity of information flow inside cells. This work is providing fundamental insight into how cells function. Additionally, it may lead to the design of novel antibiotics that disrupt the signaling of pathogenic bacteria or it could help to guide the treatment of cancer, which often involves information-processing gone awry inside human cells.

## Abstract 2

*PLOS Biology*'s first ever Research Matters explains the importance of understanding the transmission and interpretation of complex biological signals by our own cells and by the bacterial cells that live in or on us.

There are nearly 10^13^ cells in every human, and at least as many bacterial cells living in or on us [1]. Each of these cells, human and bacterial, is a sophisticated, information-processing device. Cells have evolved the remarkable ability to appraise their internal and external environments and then to act on the information gathered. They can decide whether to stay where they are or crawl away, whether to grow or hunker down until conditions improve, whether to produce one enzyme or another, and so much more. The ability to make decisions at the cellular level is absolutely critical to the survival and long-term proliferation of organisms throughout the biosphere—but how do individual cells accomplish this feat without the luxury of a brain or nervous system? The answer lies with a diverse and important set of molecules found inside all cells called signal transduction proteins [2].

These signaling proteins do not typically carry out a specific metabolic process on their own or directly participate in the growth or maintenance of cells. Instead, their job is to effectively keep tabs on the environment and respond to various cues or stimuli by activating (or inactivating) the appropriate cellular processes. Signaling proteins are, in effect, pulling the puppet strings that enable cells to survive, grow, and reproduce.

The sequencing of genomes from many different species in the late 1990s and early 2000s offered the first comprehensive assessment of the arsenal of signaling proteins available to individual cells. The signaling proteins encoded by most organisms often number in the hundreds but typically belong to a small number of protein families. The individual members of a given family are sometimes highly related at the sequence and structural levels.

In many ways, the observation that cells harbor only a small number of signaling protein families makes sense. Over the course of evolution, cells must expand and diversify their information-processing capabilities to respond to new environments and new signals. It is much easier for cells to duplicate and then modify an existing signaling modality than it is to create a brand new form of signaling protein from scratch. But the benefit of expanding an organism's signaling repertoire through duplication comes at a significant cost: how do individual cells keep signals straight and avoid unwanted cross-talk? How is specificity ensured to maintain the fidelity of information flow inside cells?

A reasonable analogy here is the telecommunication network we each rely on every day to interact with one another. For example, if I want to call my mother, I need some way to make sure my cell phone connects with her cell phone, without crossing lines or inadvertently calling someone else. This specificity is dictated by the unique phone number I enter. Is there an equivalent system, or code, used by signaling proteins to ensure their specificity?

My lab set out to address this question many years ago in bacterial cells, which rely on so-called two-component signaling pathways to perform many of their most complex information-processing tasks [3,4]. These signaling pathways involve one protein, called a histidine kinase, that resides in the membrane surrounding a cell and "listens" to the environment (Fig 1). If a signal or stimulus registers on the extracellular portion of the protein, the intracellular portion of the histidine kinase protein responds by grabbing a phosphate from ATP and attaching it to a particular histidine amino acid, a process called autophosphorylation. The kinase then docks with a second protein, called a response regulator, and passes the phosphate group to it. This regulatory protein is subsequently released to effect cellular changes, often by turning on a battery of genes that help cells cope with the environmental change or stimulus originally detected by the kinase. But how does a kinase "know" which response regulator, or substrate, to dock with and signal to?

**Fig 1:**
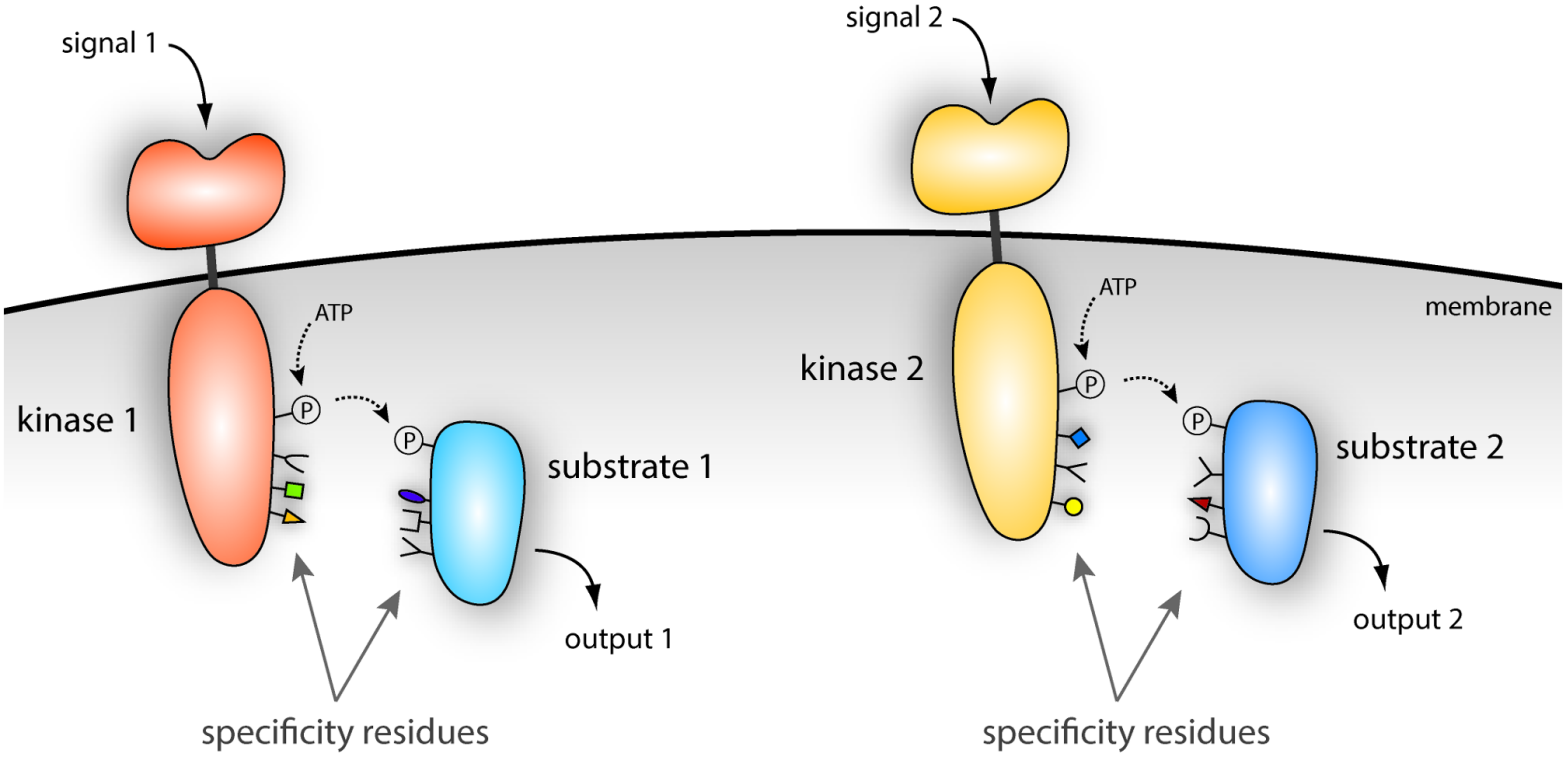
Specificity of signaling pathways. Two pathways are shown. Each initiates with a sensor kinase (orange) that can sense an extracellular signal and respond by phosphorylating itself using ATP. The phosphate group from ATP (circled 'P') can then be passed to a substrate (blue), typically a regulatory protein that can effect changes in cellular behavior. Critical to the fidelity of information flow through these pathways are a set of 'specificity residues' on each protein that are matched such that a kinase signals only to the correct substrate.

We showed that this choice, or partner specificity, is intrinsic to the kinase, meaning that the kinase has an innate ability to discriminate between the right partner and all other possible substrates, without relying on other factors inside cells [5]. This exquisite specificity is ultimately determined by a small number of amino-acid residues in the kinase located at positions in the protein near the phosphorylated histidine [6]. Each kinase has a unique set of residues at these key positions that enable it to interact exclusively with its partner, or cognate substrate, which contains a complementary set of residues (see Fig 1). Together, these paired residues (called specificity residues) in kinases and substrates form a code that ultimately ensures signals are transmitted properly inside cells.

Why do we care how signals get passed inside bacteria? Although my own lab's work on this topic is driven mainly by a curiosity about how bacteria process information, this work has several potential applications. First, it turns out that many bacterial pathogens rely on two-component signaling proteins to infect humans, so a deeper understanding of how these proteins work may enable the design of novel antibiotics that target them [7]. Like cyberattacks that seek to disrupt computer-based information networks, drugs that disrupt the information-processing of pathogenic bacteria could cripple them. Understanding the basis of signaling specificity may also enable efforts to rationally engineer bacteria as biosensors [8]. As already noted, bacteria use histidine kinases to sense and respond to a spectacular diversity of molecules and compounds in their environments. By understanding how they signal in response to these various stimuli, we can now reprogram these histidine kinases to respond in novel ways, *e*.*g*. by producing an indicator of signal detection, such as light or fluorescence.

The intrinsic and exquisite specificity of signaling proteins is, by no means, exclusive to bacteria. Exciting recent work has revealed that human kinases are also highly selective, using a defined set of specificity residues to ensure that they only phosphorylate the right substrate(s) inside cells [9]. Disrupting or altering this specificity could, in some cases, have catastrophic consequences for humans. For example, some types of cancer involve mutations in the specificity residues of signal-transducing kinases [10]. These mutations may be wreaking havoc on the information-processing capabilities of cells, possibly contributing to the unregulated growth and proliferation that is a hallmark of cancer. Thus, a better understanding of how signaling proteins ensure the specificity of their interactions may provide routes to new diagnostics or therapeutic strategies for the treatment of cancer.

Whether any of these applications in biosensing or the treatment of bacterial infections and cancer ever come to fruition remains to be seen. Regardless, future efforts in this area promise to reveal the fundamental basis of information-processing in individual cells, a phenomenon that ultimately underlies the success and diversity of almost all life on the planet.

## References

[1] Sender R, Fuchs S, Milo R. Are we really vastly outnumbered? Revisiting the ratio of bacterial to host cells in humans. Cell. 2016;164(3):337–40. 10.1016/j.cell.2016.01.013 26824647

[2] Lim WA, Mayer B., Pawson T. Cell Signaling: Garland Science; 2014.

[3] Capra EJ, Laub MT. Evolution of two-component signal transduction systems. Annu Rev Microbiol. 2012;66:325–47. 10.1146/annurev-micro-092611-150039 22746333 PMC4097194

[4] Laub MT, Goulian M. Specificity in two-component signal transduction pathways. Annu Rev Genet. 2007;41:121–45. 10.1146/annurev.genet.41.042007.170548 . 18076326

[5] Skerker JM, Prasol MS, Perchuk BS, Biondi EG, Laub MT. Two-component signal transduction pathways regulating growth and cell cycle progression in a bacterium: a system-level analysis. PLoS Biol. 2005;3(10):e334 10.1371/journal.pbio.0030334 16176121 PMC1233412

[6] Skerker JM, Perchuk BS, Siryaporn A, Lubin EA, Ashenberg O, Goulian M, et al Rewiring the specificity of two-component signal transduction systems. Cell. 2008;133(6):1043–54. 10.1016/j.cell.2008.04.040 18555780 PMC2453690

[7] Gotoh Y, Eguchi Y, Watanabe T, Okamoto S, Doi A, Utsumi R. Two-component signal transduction as potential drug targets in pathogenic bacteria. Curr Opin Microbiol. 2010;13(2):232–9. 10.1016/j.mib.2010.01.008 . 20138000

[8] Checa SK, Zurbriggen MD, Soncini FC. Bacterial signaling systems as platforms for rational design of new generations of biosensors. Curr Opin Biotechnol. 2012;23(5):766–72. 10.1016/j.copbio.2012.05.003 . 22658939

[9] Creixell P, Palmeri A, Miller CJ, Lou HJ, Santini CC, Nielsen M, et al Unmasking determinants of specificity in the human kinome. Cell. 2015;163(1):187–201. 10.1016/j.cell.2015.08.057 26388442 PMC4644237

[10] Creixell P, Schoof EM, Simpson CD, Longden J, Miller CJ, Lou HJ, et al Kinome-wide decoding of network-attacking mutations rewiring cancer signaling. Cell. 2015;163(1):202–17. 10.1016/j.cell.2015.08.056 26388441 PMC4644236

